# Gamma-aminobutyric acid-B limbic encephalitis and asystolic cardiac arrest: a case report

**DOI:** 10.1186/s13256-017-1520-z

**Published:** 2017-12-29

**Authors:** Christopher A. Ovens, Angelo Jayamanne, Andrew Duggins

**Affiliations:** 1 Nepean Hospital, Penrith, Australia; 2 Nepean Hospital Neurology Department, Penrith, Australia

**Keywords:** GABA_B_ receptor, Limbic encephalitis, Autoimmune, Paraneoplastic, Anti-Hu, Asystole, Cardiac arrest, Cardiac dysrhythmias

## Abstract

**Background:**

Gamma-aminobutyric acid-B receptor autoantibodies are becoming an increasingly recognized contributor to the spectrum of autoimmune limbic encephalitis. They are classically associated with seizures and behavioral disturbance, and may coexist with other autoantibodies. Many are paraneoplastic, most commonly associated with small cell lung cancer. Until now there have been no reports of cardiac dysrhythmias in these patients.

**Case presentation:**

A 65-year-old Caucasian man presented with multiple seizures, dysarthria and behavioral disturbance of unclear etiology, with associated asystolic cardiac arrest. Antibody testing showed anti-Gamma-aminobutyric acid-B receptor and anti-Hu antibodies in serum and Gamma-aminobutyric acid-B receptor autoantibodies in cerebrospinal fluid. The diagnosis of small cell lung cancer was subsequently made after lung biopsy, and the patient showed improvement with chemotherapy and intravenous immunoglobulin.

**Conclusions:**

We present the case of a patient with Gamma-aminobutyric acid-B receptor limbic encephalitis associated with asystolic cardiac arrest, an association not previously described. This case illustrates how difficult it is to make the diagnosis on clinical grounds alone. We therefore propose more routine antibody testing in patients with similar symptomatology who remain undifferentiated after initial workup. We also recommend that in the acute setting, patients with Gamma-aminobutyric acid-B receptor encephalitis should receive cardiac monitoring, as further research is required to clarify its possible link with cardiac dysrhythmias.

## Background

The Gamma-aminobutyric acid-B receptor (GABA_B_-R) is a metabotropic G protein-coupled receptor expressed on the surface of neurons within the central nervous system. GABA_B_-R autoantibodies are becoming an increasingly recognized contributor to the broad scope of autoimmune limbic encephalitis. They are associated with a clinical syndrome of seizures, memory impairment and behavioral changes, often in the context of small cell lung cancer (SCLC). GABA_B_-R antibodies may also coexist with other autoantibodies in patient serum and cerebrospinal fluid (CSF). We present the case of a patient with paraneoplastic anti-GABA_B_-R and anti-Hu-positive limbic encephalitis, with atypical symptomatology and an associated asystolic cardiac arrest.

## Case presentation

A 65-year-old, right-handed Caucasian man was initially admitted to another hospital after a motor vehicle accident. Prior to the accident, he was an independent truck driver who lived with his wife. He was an ex-smoker of 50 pack-years, and had a significant family history in first-degree relatives of lung, brain, and cervical cancer. Other medical conditions included hypertension, psoriasis, and diverticular disease requiring bowel resection.

The patient was driving a truck alone when he crashed. When paramedics attended, the patient was found in the passenger seat, conscious but confused and combative. At this time, pulse and blood pressure were unmeasurable. Primary and secondary surveys in hospital showed no evidence of chest trauma, and the patient suffered only minor soft tissue injuries. In hospital telemetry revealed paroxysmal atrial fibrillation with rapid ventricular response, which was without symptoms and managed only with metoprolol – to the best of our knowledge, no other antiarrhythmic agents were used. Occasional 5-second sinus pauses were also noted, with preceding seizure activity and post-ictal altered level of consciousness for several minutes. Between events, electrocardiography (ECG) was otherwise unremarkable, with no evidence of ischemic changes or other conduction abnormalities. On the fourth day of admission, he became bradycardic and progressed to asystolic arrest requiring 4 minutes of cardiopulmonary resuscitation (CPR). Spontaneous circulation returned in the form of rapid atrial fibrillation. The patient was intubated, and had a temporary pacing wire inserted until a permanent pacemaker was inserted the next day. Cardiac workup, including troponin and electrolyte levels were within normal range. Echocardiography showed a mildly dilated left atrium of 25 cm^2^, with no other valvular, structural or wall motion abnormalities noted. There was no evidence of right heart strain on echocardiogram or ECG suggestive of pulmonary embolus. A diagnosis of sick sinus syndrome was made, and he was commenced on metoprolol and apixaban. His behavior remained impulsive after extubation, demanding to leave the hospital, and he was discharged several days later. All other investigations at this time, including chest X-ray, electroencephalogram (EEG) and a computed tomography (CT) brain scan, were unremarkable.

One week after discharge, he presented to our hospital with his first observed generalized tonic-clonic seizure (GTCS) lasting 3 minutes, with urinary incontinence and prolonged post-ictal confusion. Repeat EEG and CT brain were reported as normal. This episode was thought to be secondary to hypoxic brain injury after asystolic arrest. He was discharged on levetiracetam 1 g twice daily. Two weeks later he presented with another GTCS, and was discharged once stable from Emergency. His third seizure occurred 6 days later, at which time he was admitted and commenced on sodium valproate 500 mg twice daily in addition to levetiracetam. His EEG and CT brain scan were again normal. Two weeks later, his fourth GTCS prompted addition of carbamazepine 200 mg controlled release twice daily. An outpatient magnetic resonance imaging (MRI) brain scan showed no abnormality at this time.

He had three further seizures in 3 weeks, and tolerated several antiepileptic drugs poorly. He was admitted to our hospital 10 weeks after his first seizure due to confusion. Retrospectively, there was significant deterioration in his confusion, agitation, and impulsivity since his initial presentation, in addition to more frequent seizures. Of note, his wife also reported progressive slurring of his speech, difficulty walking, and ongoing back pain over the preceding weeks. Medications at this point were lacosamide 100 mg twice daily, and sodium valproate 1 g twice daily. He suffered no further seizures, but became agitated and aggressive, requiring one-to-one nursing, regular olanzapine, and four-point limb restraints.

His refractory and progressive symptoms prompted further investigation. EEG was again normal. Lumbar puncture showed normal opening pressures, with CSF findings as follows: leukocytes 2 × 10^6^/L, mononuclear cells 2 × 10^6^/L, protein 0.61 mg/dL, glucose 3.2 mmol/L. ANNA-1 (Hu) antibodies, GABA_B_-R antibodies and unmatched oligoclonal bands were also present. Serum was also positive for GABA_B_-R antibodies but not for anti-Hu antibodies. A chest CT scan revealed a spiculated mass in the right lung and perihilar lymphadenopathy. A biopsy was obtained via endobronchial ultrasound, which revealed small cell neuroendocrine tumor. A subsequent MRI spine scan revealed diffuse vertebral metastases.

He was commenced on etoposide and carboplatin, as well as a trial of intravenous immunoglobulin (IVIg). Although suffering chemotherapy-related side effects including febrile neutropenia from staphylococcal septicemia, he showed some improvement with this therapy. He remained seizure free, was able to hold a conversation and mobilise independently. His modified Rankin scale (mRS) was 4 prior to treatment, and improved to a mRS of 2 with IVIg and chemotherapy. At 12 weeks follow-up, our patient had only mild short-term memory deficits, but was otherwise at his premorbid level of function.

## Discussion

We report a case of a patient with anti-GABA_B_-R and anti-Hu limbic encephalitis, presenting with progressive seizures and behavioral disturbance, as well as cardiac dysrhythmias. While much of his phenotype was classic of GABA_B_-R encephalitis, phenotypic overlap is rare and an association with cardiac dysrhythmias has not previously been described. The first paraneoplastic antibodies were to intracellular targets (e.g., anti-Hu, -Ma2, -CRMP5, -amphiphysin). Anti-GABA_B_-R antibodies join the family of the more recently discovered antibodies against cell surface targets, such as anti-NMDAR, -AMPAR, -GlyR, -Caspr2, and -LGI1 [1]. All of the GABA_B_-R-positive patients described by Lancaster *et al*. [2] presented with early or prominent seizures, with the majority showing limbic dysfunction in the form of confusion, memory disturbance, and behavioral changes. This is thought to be due to the inhibitory action of GABA_B_-R with high density in the hippocampus [3, 4]. Further case series and case reports have broadened the scope of anti-GABA_B_-R syndromes [1–7]. Half were associated with SCLC, (27/53), two were associated with other cancer (thymus carcinoid, melanoma), while the remainder were not paraneoplastic. The majority were associated with seizures (46/53), however atypical phenotypes have been reported including cerebellar ataxia, opsoclonus myoclonus, brainstem encephalitis, and status epilepticus. Cerebellar ataxia and opsoclonus myoclonus may be related to high GABA_B_-R density in the cerebellum [3, 4]. MRI changes involve high signal in limbic regions on T2/FLAIR images, however MRI was normal in 32% of patients. Outcomes varied, but the majority showed some improvement with immunotherapies and oncological treatment where tumor was present. Patients with SCLC have poorer survival compared to those without tumor [3].

The presence of dual autoantibodies in our patient (anti-GABA_B_-R and anti-Hu) is notable. Only three patients have previously been described with this combination [4, 5]. Classically, anti-Hu antibodies confer a poor prognosis and are associated with sensory or sensorimotor neuropathy, however they are not always pathogenic [4, 8]. Our patient also displays phenotypic overlap. The presence of dysarthria (prominent in our patient) in GABA_B_-R encephalitis has been described only in the setting of cerebellar ataxia phenotype [3, 5] or brainstem phenotype [7]. To the best of our knowledge, dysarthria has not been described in combination with the prominent seizures and behavioral changes of classic limbic encephalitis. This overlap may be due to either anatomically extensive GABA_B_-R encephalitis (possibly with limbic and cerebellar involvement) or ‘silent’ anti-Hu antibodies, or due to anti-Hu antibodies modifying a more classical GABA_B_-R phenotype.

Two controls in a previous study [2] had low titer GABA_B_-R antibodies and high titer GAD-65 antibodies, and phenotypically resembled a GAD-65 encephalopathy with progressive cerebellar ataxia, rigidity, myoclonus, and gait instability. Similarly, Hoftberger *et al*. [3] reported a patient with GABA_B_-R and amphiphysin autoantibodies with a mixed phenotype of limbic encephalitis, diffuse encephalomyelitis and gait ataxia. The significance of antibody titers is still under investigation. Although Lancaster *et al*. [2] showed no correlation between titer and disease severity, Mundiyanapurath *et al*. [7] showed a steady decline in titer mapping treatment response. The clinical manifestation of GABA_A_-R autoimmune encephalitis is dependent on titer. High titers cause encephalitis while low titers invoke seizures, stiff-person syndrome and opsoclonus myoclonus [9]. Thus, while different individuals may have different disease thresholds, titer may help to determine symptomatology when multiple competing antibodies are present.

Our patient’s motor vehicle accident was initially thought to be a primary cardiac event, with subsequent asystole and pauses consistent with sick sinus syndrome. If this was the case, then two distinct cardiac events (in addition to other arrhythmias) preceded the development of seizure activity. It is known that elevated levels of S100B, a dimeric calcium-binding protein in brain astrocytes, has been associated with conditions of hypoxia, such as high altitude and obstructive sleep apnoea [10, 11]. S100B is also believed to be a marker of blood brain barrier (BBB) dysfunction [11]. In addition to hypoxia, acute stress, and epinephrine have also been shown to alter BBB permeability [10]. If the hypoxia or acute stress from cardiac arrest preceded neurological symptoms, it is conceivable that this BBB disruption may have granted serum GABA_B_-R autoantibodies access to cerebral tissue. This would also allow translocation of activated memory B cells into the central nervous system [10], mediating subsequent intrathecal production of autoantibodies similar to the mechanism proposed in multiple sclerosis [12]. However, the fact that our patient’s first documented seizure occurred 1 week after his accident casts doubt over this hypothesis, requiring a very rapid humoral response within the central nervous system. It also fails to identify a cause for his sinus dysfunction. Our patient had no history or family history of cardiac disease or sudden death. He had suffered no previous episodes of palpitations, chest pain, or syncope. Aside from a mildly dilated left atrium, there were no other cardiac abnormalities detected on routine workup, and no underlying precipitant for his sick-sinus syndrome was found.

In retrospect, we believe our patient’s initial motor vehicle accident to instead be secondary to a seizure. That he was found in the passenger seat raises the possibility of convulsions, and if the force of impact alone were enough to throw him into the passenger seat, we would expect further injuries to be seen on his secondary survey. His agitation and confusion at the scene are consistent with a post-ictal phase. An ictal or post-ictal asystolic event would explain the non-palpable pulses and unrecordable blood pressure at the time of his motor vehicle accident, as well as the sinus pauses on telemetry associated with seizure activity. Ictal and post-ictal conduction abnormalities are rare but well-described complications of seizures, with asystole, bradycardia, and other arrhythmias potentially involved in the pathogenesis of sudden unexplained death in epilepsy patients (SUDEP) [13–15]. In asystole, it is thought that seizure activity disrupts temporal and insular connections with the brainstem and hypothalamus, causing a transient excess of vagal tone [13, 14]. Ictal asystole is classically brief (90% lasting less than 30 seconds) and is always associated with a focal seizure, with or without secondary generalization. Progression to GTCS is associated with prolonged ictal asystole (up to 96 seconds has been reported) [14]. In post-ictal asystole from generalized seizures, the greatest reported delay between seizure and onset of asystole was 158 seconds, asystole duration was 7–60 seconds, and more than half died from probable SUDEP [15]. However, the patient’s in-hospital asystolic cardiac arrest would be atypical for ictal asystole, lasting more than twice as long as those previously described and lacking preceding seizure activity.

Thus, while it is possible that isolated cardiac dysrhythmias precipitated neurological symptoms, we believe this mechanism to be less likely due to the events surrounding his initial motor vehicle accident, its time course, and the absence of other cardiac abnormalities as a cause. His asystolic arrest is also insufficiently explained purely by a post-ictal mechanism, and as a result we propose a link between our patient’s underlying GABA_B_-R encephalitis and his cardiac dysrhythmias and asystole. Other encephalitides have been associated with asystole and cardiac arrhythmias, including N-methyl-D-aspartate receptor (NMDA-R) autoimmune limbic encephalitis [16, 17] and viral encephalitis [18]. In one series of 100 patients more than 1/3 of patients with NMDA-R encephalitis had cardiac dysrhythmias in addition to the classical symptoms of psychiatric and behavioral disturbance – 7% had prolonged pauses and 4% required pacemaker insertion [16]. In this series, it is unclear whether these were associated with seizures or not. A smaller series [19] identified 80% of NMDA-R encephalitis patients with tachycardias, 60% with sinus bradycardias and 40% with sinus arrests. The authors suggest that NMDA-R dysfunction within the nodose vagal nuclei and the nucleus of solitary tract (NTS) lead to abnormal vagal responses capable of tachyarrhythmias, bradycardias, and asystole. GABA_B_-Rs have been shown to populate similar areas of the brain including the NTS and vagal nuclei [19–21]. They have similarly been implicated in modulating vagal cardiorespiratory reflexes in animal models [20, 22, 23]. It is possible that in our patient, a similar disruption of vagal reflexes by GABA_B_-R antibodies may account for his sinus node dysfunction, pauses, and asystolic arrest. Interestingly, Nazif noted a progressive bradycardia and subsequent arrest in vagally mediated NMDA-R asystole, and a similar progression was seen in our patient [19]. However, it is unclear whether a significant vagal stimulus was present prior or not. This mechanism of autonomic dysregulation and vagal disturbance has also been proposed to explain ictal asystole, and may represent a common pathway in these patients [13, 14]. Thus we propose an association between GABA_B_-R limbic encephalitis and cardiac dysrhythmias, which has not previously been described.

## Conclusions

In summary, we present a 65-year-old man with anti-GABA_B_-R and anti-Hu paraneoplastic limbic encephalitis secondary to small cell lung cancer, with associated asystolic cardiac arrest. The cause for this patient’s arrhythmias, seizures, and confusion was initially obscure, and remained so even after imaging and routine blood workup. The initial diagnosis of hypoxic brain injury was called into question by the progressive nature of his symptoms, his dysarthria and ongoing back pain. Given the diverse phenotypes associated with cell surface autoimmune encephalitides, and given that clinical outcome depends on early diagnosis in these conditions (especially when associated with tumor), a case can be made for more routine screening in patients who show progressive and refractory neurological symptoms and who remain undifferentiated after initial workup. In light of this case, telemetry is also warranted in those patients with confirmed GABA_B_-R encephalitis to further clarify any association with cardiac dysrhythmias.

## Abbreviations

BBB: Blood brain barrier
CSF: Cerebrospinal fluid
CT: Computed tomography
ECG: Electrocardiography
EEG: Electroencephalogram
GABA_B_-R: GABA_B_ receptor
GTCS: Generalized tonic-clonic seizure
IVIg: Intravenous immunoglobulin
MRI: Magnetic resonance imaging
NMDA-R: N-methyl-D-aspartate receptor
SCLC: Small cell lung cancer
SUDEP: Sudden unexplained death in epilepsy patients
